# Charity and Commercialism

**Published:** 1920-03-13

**Authors:** 


The Hospital

The Workers' Newspaper of Administrative Medicine and Institutional Life, Administration, National Insurance and Health.

No. 1762, Vol. LXVII. SATURDAY, MARCH 13, 1920. PRICE TWOPENCE

### CHARITY AND COMMERCIALISM.

The correct method of financing a voluntary hospital is to steer between two excesses, each of which after periodic indulgence here or there produces a reaction toward its opposite. When the hospitals are acutely short of money, and recent methods of direct appeal seem to be losing their effect, the more spry among the younger men begin to resort to the latest shifts of commerce. Their attitude has been simplified by an acute consciousness of the need for money, and this consciousness provokes them to engage in any enterprise that seems to have been found lucrative by commercial astuteness. Perhaps exhibitions are the rage at the moment. If so the eager administrator will endeavour to earn the thanks of his committee by organising monster bazaars, café chantants, and the like, or even a gala performance at a music-hall. These methods often succeed in raising considerable sums, but the man who is content with them soon discovers that the public palate is fickle, and must be tickled ever afresh. He has employed a palliative as if it were a policy, and, getting desperate, the type which we have in mind is apt to verge upon proceedings which are not merely commercial, but intrinsically objectionable. Before that temptation becomes too powerful he is sometimes saved by some emergency, which creates a new but recognised way of raising funds. The war was one of these emergencies and encouraged, though it did not create, the method of street collecting on a scale and with an organisation never attempted before. The vogue and recoil from the unlimited flag day is typical.

Now the commercial method of raising money for a voluntary hospital is always recognisable by the application of two tests. The first is the element of sale; when people do not give voluntarily, but they can be induced to give by receiving in exchange something of value: an entertainment, a luncheon, music, or a badge. Where there is such an exchange the commercial element is obvious. The second test of the commercial element in an "appeal" is the reduction of the voluntary motive to vanishing point, and the surreptitious substitution in its place of a more or less veiled compulsion. The house-to-house collection, in tactless hands, verges towards compulsion; solicitation in the street, with or without the "purchase" of a badge, is compulsion unashamed. In fact the flag became popular because it kept the female pest away. In all these cases the object of the appeal is lost in the method adopted to sustain it.

If that obvious fact is considered attentively, even for a few moments, it will be evident that the voluntary system cannot endure if its native principle is to be invaded by perpetual resort to methods which are the antithesis of the principle itself. Let there be no mistake. The hospitals will remain with us, but they need not be voluntary hospitals, nor will the voluntary spirit continue to permeate them if the voluntary system is discarded. It is, therefore, worth noting that at the moment when resort to commercial methods has reached its climax and has been found to break in the hands of those who trusted to it most, the cry for State control was immediately raised. Our point is that this cry was a necessary consequence, and that the people who began by preferring commercial to voluntary methods of raising money ended by preferring even to commercial methods the still more "business-like" sustenance of the State.

Having discovered precisely where we are in this matter, let us turn to an example of the reaction which, we began by stating, always succeeds such an excess. Mr. Francis Morris, chairman of the Administrative Committee of the C.O.S., lately complained in *The Times* of certain "undignified methods of appeal." He pointed out that rapid and sensational methods of raising money, which were excusable during the war, are "inappropriate to a complex and permanent system of voluntary charity." He quoted various undesirable instances, and rightly insisted that "the serious purposes of charity are obscured by methods of this kind," which also lead to several practical abuses. It was lack of space, presumably, which prevented Mr. Morris from proceeding to suggest a remedy.

The real problem at the moment is not to raise money for the hospitals, since the State could supply that in the last resort, but to maintain the voluntary system by voluntary means. This can be done only by men who believe in the voluntary principle as the principle, who prefer voluntary means to any other, and who are sincere enough to infect others with their own belief. Do such people still exist? We are sometimes tempted to wonder as we scan the crowd of doubting Thomases upon which many a hospital now seems to repose. Were there really an absence of such believers the case would be hopeless. Principle, conviction, belief in the doctrine of personal service as the backbone of the voluntary system, this alone has the power to subdue all things to itself. This alone secures that a hospital's work shall be its own best advertisement, that publicity is not an end but a means, that the public shall be interested because a hospital is the living centre of its own area. At this moment a man with that conviction has an instrument to hand such as he will not meet again in his life. The spirit of voluntary effort, which the hospitals had nursed, received a new impetus from the war. It touched those who had not been touched before. It reached those beyond its previous influence. Many of these found their shepherd in the Red Cross Society, which was represented by a centre of energy in every county, in every town, in many a village even. These centres did work, made articles, gave time and labour. The money was mostly collected apart from them. The Red Cross is now trying to continue these local centres for the benefit of the local hospitals. How far the plan will be successful depends upon the response which these centres receive from the men at the hospital to which they are affiliated and the extent to which they are something much more than a body of collectors of local funds. If the right men are found the methods will take care of themselves, for they will express the spirit of their authors. Let us see to it that Personal Service, not mere publicity, remains the chief energy to which we trust.

